# hiPSC-derived new approach methodology (NAM) recapitulates hallmarks of atrial fibrillation

**DOI:** 10.3389/fcvm.2026.1814075

**Published:** 2026-10-20

**Authors:** Paul Kozlowski, Kalai Mangai Muthukumarasamy, Tiago William Buranello, Sung Jo, Amrish Deshmukh, Andre Monteiro da Rocha, Hakan Oral

**Affiliations:** 1 Medical School, University of Michigan, Ann Arbor, MI, United States; 2 Internal Medicine – Cardiovascular Medicine, University of Michigan, Ann Arbor, MI, United States; 3 Frankel Cardiovascular Center Cell Regeneration Core Laboratory, University of Michigan, Ann Arbor, MI, United States

**Keywords:** atrial cardiomyocyte, atrial fibrillation, atrial fibroblast, electrophysiology—optical mapping, fibrosis, hiPSC-CM, intermittent tachypacing, new approach methodologies—NAMs

## Abstract

**Background and aim:**

Human *in vitro* models for atrial fibrillation (AF) remain limited in reproducing disease-specific remodeling. We leveraged a human-induced pluripotent stem cell-derived atrial cardiomyocyte (hiPSC-aCM) model, which was co-cultured with human atrial cardiac fibroblasts (haCFs) and subjected to an intermittent tachypacing protocol (ITPP) to mimic AF-associated phenotypes.

**Materials and methods:**

Human iPSC-derived atrial cardiomyocytes (hiPSC-aCMs) were differentiated, purified, matured, and cultured alone or co-cultured with human atrial cardiac fibroblasts at defined seeding ratios (e.g., 90/10 or 70/30 hiPSC-aCM/haCF). Cultures were subjected to 7 days of intermittent tachypacing using electrical field stimulation. Functional electrophysiology and calcium handling were assessed by high-throughput optical mapping, arrhythmia inducibility testing, and genetically encoded or chemical calcium indicators. Structural and molecular remodeling were evaluated by immunocytochemistry, qPCR, and Western blot analysis.

**Results:**

ITPP increased spontaneous action potential (AP) frequency and shortened AP duration at 80% repolarization (APD_80%_), while reducing calcium transient (CaT) upstroke slope and amplitude. Co-cultures at a 70/30 hiPSC-aCM/haCF ratio exhibited marked structural remodeling, with a >100-fold increase in Collagen III expression (*P* < 0.0001), decreased excitability (ΔHz = –61 ± 6%, *P* < 0.0001), prolonged APD_80%_ (Δ = +130 ± 10%, *P* < 0.0001), increased triangulation (ΔAPD_Tri_ = +143 ± 13%, *P* < 0.0001), reduced upstroke slope (Δ = –66 ± 6%, *P* < 0.0001), conduction block (Δ = –52 ± 18%, *P* = 0.026), and impaired Ca^2^⁺ handling (upstroke slope Δ = –50 ± 8%, *P* < 0.0001; ΔdF/F0 = –34 ± 9%, *P* = 0.0003). Applying ITPP to the 70/30 co-culture induced AF-like remodeling, with increased spontaneous activity (ΔHz = +25 ± 8%, *P* = 0.02), shortened APD_80%_ (Δ = –16 ± 6%, *P* = 0.01), and reduced conduction velocity (ΔCV_100/0 vs. 70/30_ = –27 ± 15%, *P* = 0.0005). Mechanistically, ITPP led to downregulation of phospholamban (PLN) and altered PLN-SERCA2a balance, along with upregulation of TGFB1 and natriuretic peptides, reflecting calcium-handling dysfunction and pro-fibrotic signaling.

**Conclusion:**

Together, these findings establish a human-relevant platform in 2D culture that recapitulates key AF-associated structural and electrophysiological remodeling for mechanistic studies and therapeutic screening.

## 1 Introduction

The gap in translation between animal models and human diseases has led to recent changes in policy and funding including the New Approach Methodologies (NAMs): the FDA Modernization Act 2.0 (2022) (1) and other initiatives such as Complement-ARIE Common Fund program to accelerate human-relevant NAMs (NIH Complement-ARIE) (2) and the recent establishment of the NIH Center for Standardized Organoid Modeling (SOM) in 2025 (3).

A variety of small and large animal models have been utilized to study atrial fibrillation (AF) for decades (4). Nevertheless, challenges in extrapolating findings from animal models to humans, and limitations in current model organisms and experimental methods hinder the translation of scientific discoveries into clinical treatments (5). AF remains the most prevalent cardiac arrhythmia with lifetime risk exceeding 30% (6); and although both pharmacological interventions and invasive procedures are capable of delaying the progression of AF, recurrence rates nonetheless range between 46% and 59% (7, 8).

In this context, human pluripotent stem cells (hPSCs) are an emerging New Approach Methodology valuable for personalized medicine, drug discovery (9–14), and the study of cardiac genetic and acquired diseases (15–19). In order to design a NAM that recapitulates key features of AF (electrical and structural remodelling), we leveraged co-culture of human induced pluripotent stem cell-derived atrial cardiomyocytes (hiPSC-aCM) (20–22) with human adult atrial cardiac fibroblasts (haCF) alongside a well-studied human perinatal stem cell derived extracellular matrix (ECM) that promotes structural, metabolic and functional maturation in hiPSC-CMs (21–23) and intermittent tachypacing to reproduce *in vitro* electrical and structural remodelling associated with AF. This novel, modular NAM model captures key features of AF by recapitulating electrical and intracellular calcium-handling remodeling and endomysial fibrosis, which aims to serve as a valuable testbed for advancing therapeutic approaches in atrial fibrillation.

## 2 Materials and methods

### 2.1 Intermittent tachypacing of hiPSC-aCM and hiPSC-aCM/haCF co-culture

Human iPSC-aCMs differentiated (24) from DF19-9-11T human iPSC line (WiCell Research Institute) and matured as previously described (21, 22) were co-cultured with varying percentages of haCFs (Lonza, #CC-2903) to accommodate 100%, 90%, or 70% hiPSC-aCM. For pacing experiments, the cells were plated in 8-well plates coated with MatrixPlus (StemBioSys) and fitted with a polydimethylsiloxane (PDMS) silicone sheeting (Specialty Manufacturing, Inc, Saginaw, MI) imprinted with a circle (3.14 cm^2^) of free space to restrict the seeding area of the cells. The cells were subjected to chronic electrical field pacing using a C-PACE EP Cell Culture Stimulator Bank (IONOptix) coupled with C-Dish™ 8-well carbon electrodes (IONOptix). An intermittent tachypacing protocol was established by applying electrical field stimulation at 20 V/cm with 0.5 ms pulse duration at frequency cycles at 1.5 Hz for 45 min followed by 5 Hz for 15 min, repeated hourly for a duration of 7 days. All experiments were performed using two independent differentiation batches, except for the immunostaining experiments shown in Figure 5, which were generated from a single batch. This study was conducted in accordance with the Human Pluripotent Stem Cell Research Oversight (HPSCRO #0147) ethical approval. The research presented in this manuscript is the subject of a pending patent application 63/761,636 (USPTO), filed by Drs. Oral and Monteiro da Rocha, and Paul Kozlowski.

### 2.2 High-throughput optical mapping

Optical mapping was performed using a CARTOX™ device (StemBioSys) at 37 °C. For mapping of calcium transients (CaTs) hiPSC-aCM monolayers were pre-loaded with the genetically encoded calcium indicator—GCaMP6f, as previously described (21). Mapping of CaTs in hiPSC-aCM/haCF co-culture preparations was facilitated using the Calbryte™ 520 AM intracellular calcium indicator (20651; AAT Bioquest). Action potentials were recorded using the FluoVolt probe (F10488; Life Technologies). Following a 30 min incubation, monolayers were washed with HBSS with calcium and magnesium, then incubated at 37 °C for 1 h prior to optical mapping. Data analysis was conducted using StemBioSys software.

Detailed description of the methods can be found in the Supplementary Material.

## 3 Results

### 3.1 hiPSC-aCMs phenotype and maturation

We subjected hiPSCs to a chamber-specific cardiomyocyte differentiation protocol previously published (21, 22, 25, 26) to compare spontaneous calcium transients, oxygen consumption rate and gene expression of hiPSC-aCMs and -vCMs. The frequency of spontaneous activation was higher in hiPSC-aCMs in comparison to -vCMs (Figures 1A,B; *p* < 0.0001) and consequently the CaTD80% in hiPSC-aCMs was significantly shorter than in hiPSC-vCMs (Figure 1C, *p* < 0.0001). The higher spontaneous activity in hiPSC-aCMs was associated with higher oxygen consumption rate in comparison to hiPSC-vCMs (Figure 1D; *p* < 0.0001).

**Figure 1:**
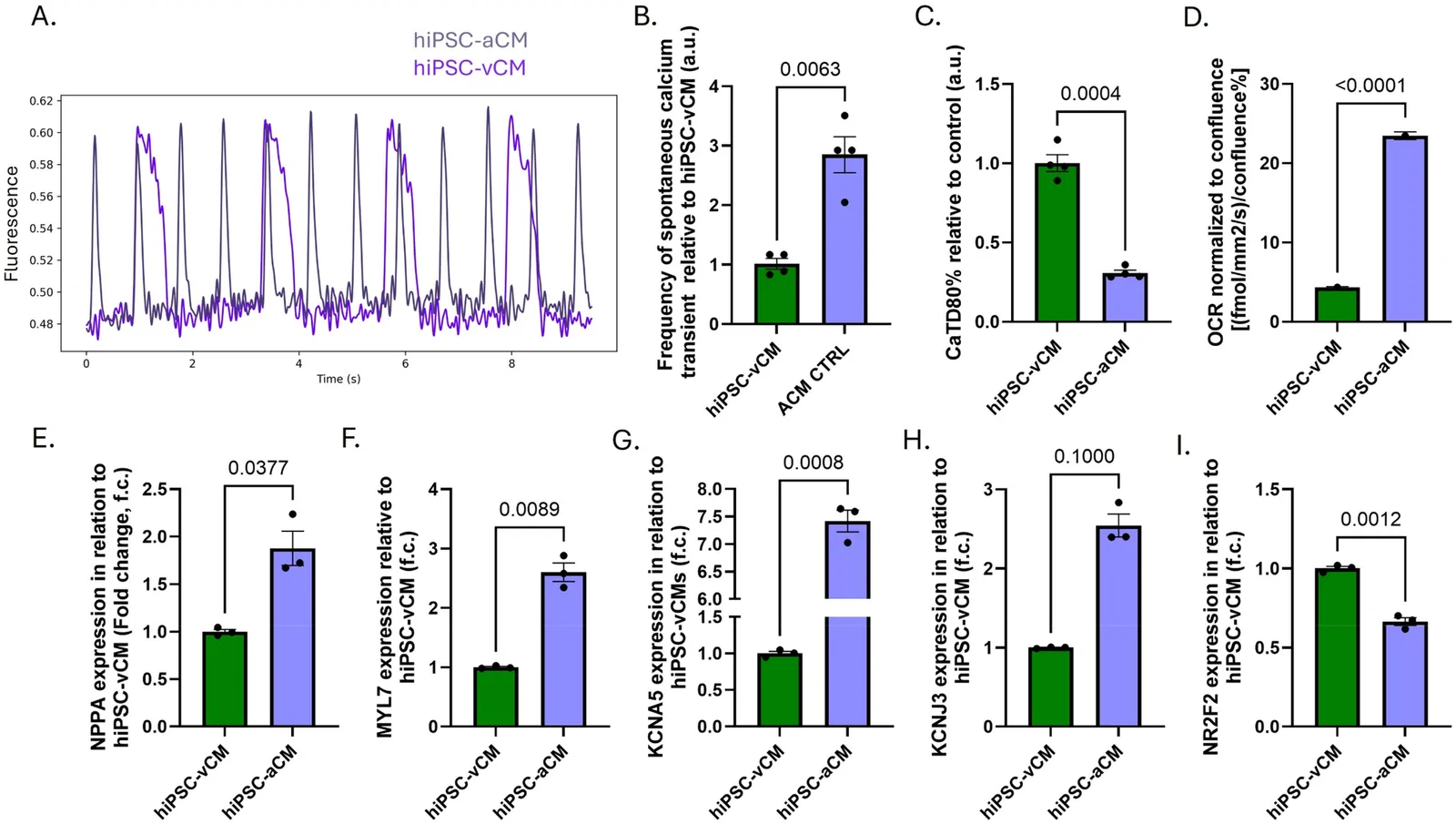
Functional comparison of hiPSC-derived atrial- and ventricular cardiomyocytes. **(A)** Representative spontaneous activity trace from hiPSC-aCMs and –vCMs indicating differences in frequency of spontaneous activation. (**B,C**) Frequency of spontaneous calcium transients is higher in hiPSC-aCMs in comparison to hiPSC-vCMs and consequently present shorter CaTD80%. **(D)** the oxygen consumption rate was higher in hiPSC-aCMs than -vCMs. The expression of **(E)** NPPA, **(F)** MYL7, and **(G)** KCNA5 were higher in hiPSCaCMs in comparison to hiPSC-vCMs. **(H)** Expression of KCNJ3 was higher in hiPSC-aCMs, although the difference was not statistically significant. **(I)** Expression of NR2F2 was significantly lower in hiPSC-aCMs in comparison to -vCMs. Across the quantified readouts, hiPSC-aCMs and hiPSC-vCMs display subtype-dependent differences in electrical activity and oxygen consumption rate, consistent with distinct atrial- and ventricular-like gene expression. Bars represent mean ± SEM, with individual data points overlaid to show biological replicates.

A subset of genes known to be differentially expressed in atrial and ventricular cardiomyocytes was assessed with qPCR. Expression of the gene encoding atrial natriuretic peptide (NPPA) was higher in hiPSC-aCMs in comparison to hiPSC-vCMs (Figure 1E; *p* = 0.037) and expression of the gene encoding myosin light chain (MYL7) was also significantly higher in hiPSC-aCMs in comparison to -vCMs (Figure 2F; *p* = 0.0089). The expression of KCNA5 and KCNJ3 were higher in hiPSC-aCMs than in -vCMs, although only the expression of KCNA5 was statistically significant (Figure 1G; *p* = 0.0008; and Figure 1H; *p* = 0.1). Nevertheless, the expression of the transcription factor encoded by NR2F2 was lower in hiPSC-aCMs than in -vCMs (Figure 1I; *p* = 0.0012).

**Figure 2:**
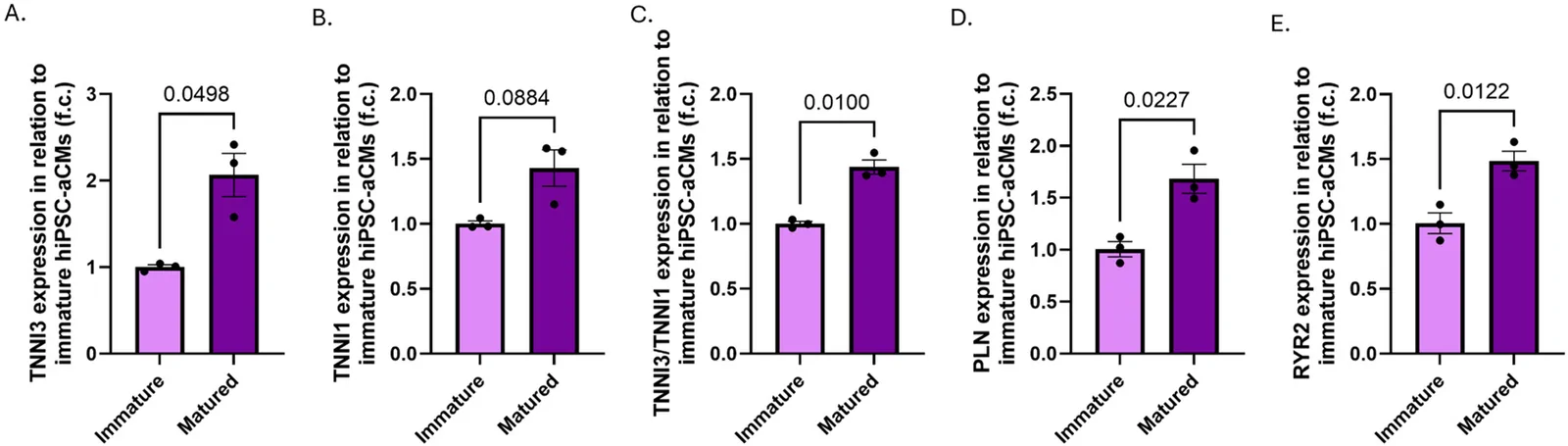
Comparison of gene expression in immature and matured hiPSC-aCMs. **(A)** Expression of the maturation marker TNNI3 was higher in matured hiPSC-aCMs in comparison to immature hiPSC-aCMs. **(B)** The expression of TNNI1 was not significantly different between matured and immature hiPSC-aCMs; **(C)** but the TNNI3/TNNI1 ratio was significantly higher in mature hiPSC-aCMs in comparison to immature hiPSC-aCMs. Further the expression of **(D)** PLN and **(E)** RYR2 were significantly higher in matured hiPSC-aCMs than immature hiPSC-aCMs.

Extracellular matrix mediated hiPSC-CM maturation has been previously described by our group (21–23, 25, 26). Herein, we assessed the expression of the genes encoding slow skeletal troponin I (TNNI1; immature marker), cardiac troponin I (TNNI3; maturation marker), the ratio between the two markers, and calcium handling genes (PLN and RYR2). The expression of TNNI3 was higher in matured than in immature hiPSC-aCMs (Figure 2A; *p* = 0.049). The expression of TNNI1 was similar between groups (Figure 2B; *p* = 0.08), however the TNNI3/TNNI1 ratio was significantly higher in matured hiPSC-aCMs than in immature (Figure 2C; *p* = 0.01). Expression of both PLN (Figure 2D; *p* = 0.022) and RYR2 (Figure 2E; *p* = 0.012) were higher in matured hiPSC-aCMs.

### 3.2 Intermittent tachypacing protocol (ITPP) induces a tachycardia-like phenotype in hiPSC-aCM syncytia

Optical mapping of action potentials (APs, Figure 3A) revealed that ITPP-treated hiPSC-aCM syncytia exhibited a significantly higher frequency of spontaneous APs relative to control (non-paced) syncytia (*P* < 0.0001; Figure 3B). Non-normalized spontaneous beating rates (not shown) composing Figure 3 consisted of Replicate 1: 100/0 Control: 0.91 ± 0.05 Hz, *n* = 6; 100/0 ITPP: 1.21 ± 0.07 Hz, *n* = 7 and Replicate 2: 100/0 Control: 2.13 ± 0.09 Hz, *n* = 7. 100/0 ITPP: 2.75 ± 0.21 Hz, *n* = 6. Changes in frequency were associated with Fridericia-corrected APD_80%_ (*P* = 0.001; Figure 3C) and AP triangulation (ΔAPD90–APD50; *P* = 0.01; Figure 3D) shortening in ITPP-treated cells, suggesting a reduction in phase 3 repolarization. The upstroke slope of the AP, a measurement of phases 0/1, was not affected by ITPP (*P* = 0.78; Figure 3E). The conduction velocity of the AP was significantly faster following ITPP (*P* = 0.0002; Figure 3F).

**Figure 3:**
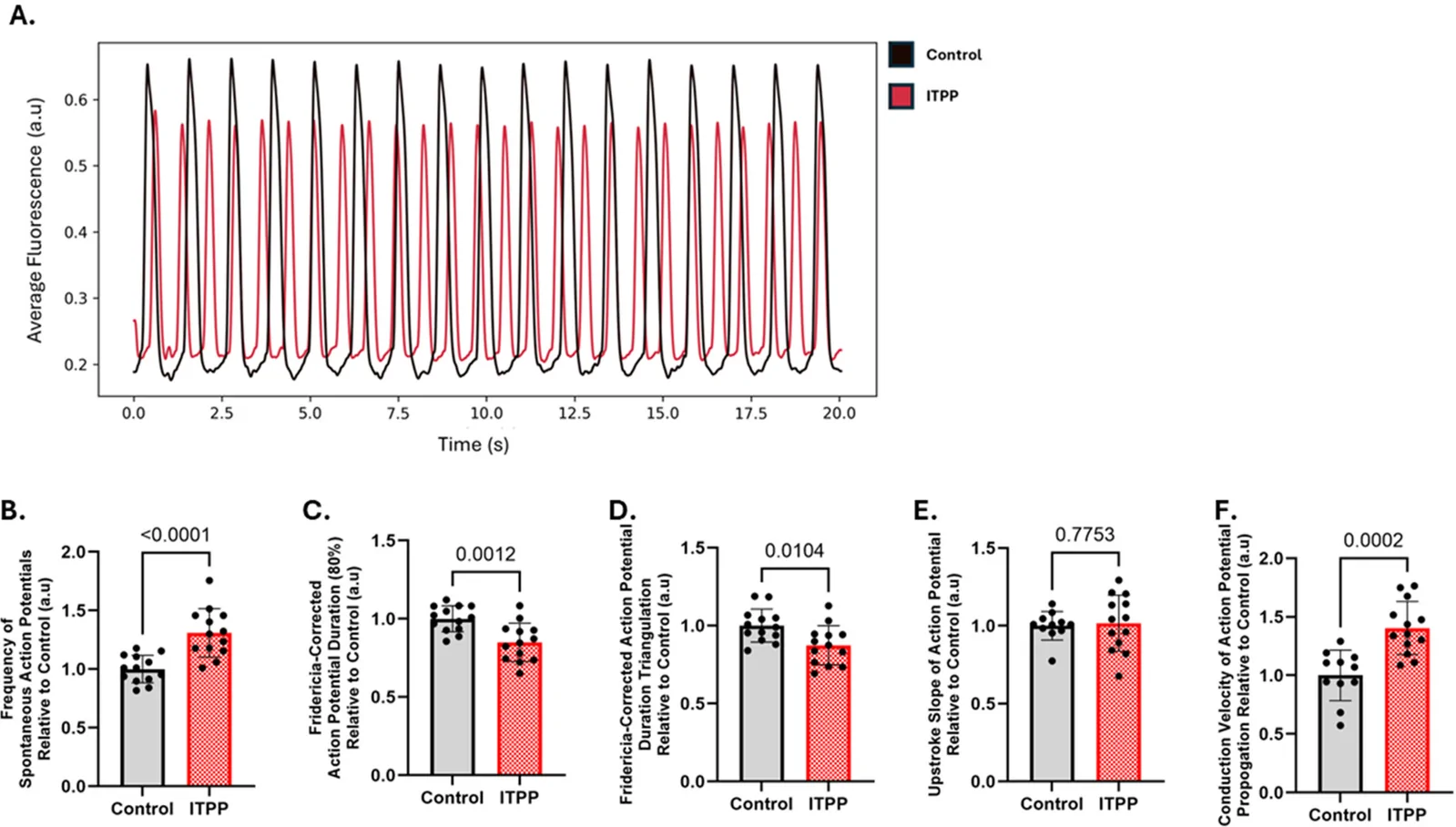
Functional electrical remodeling of hiPSC-aCM syncytia submitted to ITPP. **(A)** Time-sequence plot of representative action potential traces of hiPSC- aCM syncytia of control (black) and ITPP (red). **(B)** Paced hiPSC-aCM syncytia exhibited an increased frequency of spontaneous APs relative to control (Δ = +31 ± 7%, *p* < 0.0001) alongside reductions in Fridericia-corrected AP duration (80%) (Δ = −15 ± 4%, *P* = 0.0012) **(C)** and AP triangulation **(D)** (Δ = −13 ± 5%, *P* = 0.0104), with preserved upstroke slope **(E)** and significantly faster conduction velocity **(F)** (Δ = +41 ± 9%, *P* = 0.0002).

### 3.3 ITPP recapitulates AF intracellular calcium handling dysregulation

The effects of ITPP on intracellular calcium handling of hiPSC-aCM syncytia were evaluated by optical mapping of intracellular Calcium Transients (CaTs). Baseline frequency of spontaneous activation was not significantly different between ITPP-treated hiPSC-aCM syncytia and controls (non-paced cells; *P* = 0.16; Figure 4A); however, there was a significant increase (Δ = +332 ± 90%) in the variability of calcium release frequency following ITPP (*P* = 0.0006; Figure 4B), which was measured using the standard deviation of frequency of spontaneous CaTs.

**Figure 4:**
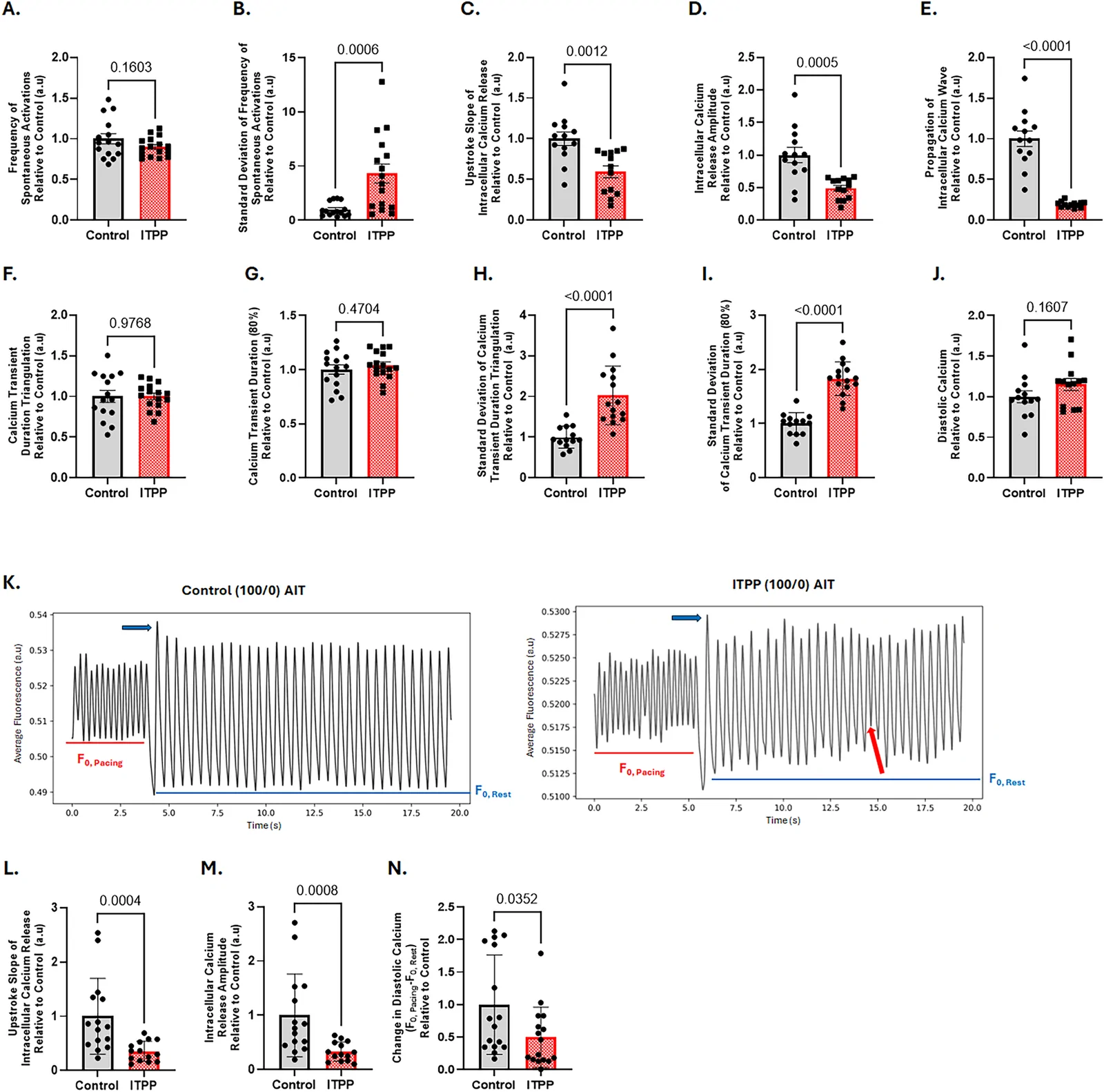
Intracellular calcium handling alterations of hiPSC-aCM syncytia subjected to ITPP. **(A)** ITPP did not influence the frequency of spontaneous calcium release but markedly increased the variability of calcium release events (Δ = +332 ± 90%, *P* = 0.0006) **(B)**. **(C)** ITPP decreased both the upstroke slope (Δ = −41 ± 11%, *P* = 0.0012) and **(D)** amplitude of intracellular calcium release (Δ = −51 ± 13%, *P* = 0.0005) with slowed propagation speed of the calcium wave **(E)** (Δ = −81 ± 10%, *P* < 0.0001). No change in average speed of intracellular calcium reuptake **(F)** and in CaTD_80%_ duration **(G)** was affected by ITPP. **(H,I)** The beat-to-beat variability of both CaTD_Tri_ (Δ = +103 ± 20%, *P* < 0.0001) and CaTD_80%_ (Δ = +83 ± 10%, *P* < 0.0001) was significantly increased. **(J)** The diastolic calcium level following the CaT remained unchanged following ITPP. **(K)** Representative traces of Arrhythmia inducibility test **(**AIT) stimulation in Control and ITPP hiPSC-aCM syncytia demonstrate transition between AIT pacing and rest. The post-rest potentiation calcium transient (PRP-CaT) marking the transition from pacing to rest is labeled with a blue arrow; a premature calcium release event is labeled with a red arrow. Diastolic calcium levels during AIT pacing (red line) and rest (blue line) are indicated. **(L)** The PRP-CaT, marking calcium flux during transition from AIT pacing to rest, presented decreased upstroke slope of intracellular calcium release (Δ = −65 ± 19%; *P* = 0.0004), **(M)** decreased intracellular calcium release amplitude (Δ = −67 ± 20%; *P* = 0.0008), and **(N)** reduced recovery of baseline calcium levels after AIT cessation (Δ = −50 ± 22%, *P* = 0.0352).

The upstroke slope of the CaT proxies the speed of calcium flux during depolarization. Compared to controls, ITPP-treated hiPSC-aCM syncytia exhibited a significantly slower upstroke slope of calcium release (*P* = 0.001; Figure 4C). Calcium release amplitude was also significantly diminished in ITPP-treated cells (*P* = 0.0005; Figure 4D), and the propagation speed of the intracellular calcium wave across the monolayer was significantly slower (*P* < 0.0001; Figure 4E). We did not observe any significant changes to CaTD_Tri_ (*P* = 0.98; Figure 4F); a proxy of the duration of intracellular calcium reuptake. The CaT at 80% of intracellular calcium recapture (CaTD_80%_) was similar between the groups (*P* = 0.47; Figure 4G). However, there was a significant increase in the beat-to-beat variability in reuptake duration (*P* < 0.0001; Figure 4H) in ITPP-treated hiPSC-aCMs compared to controls, alongside an increase in the standard deviation of CaTD_80%_ (*P* < 0.0001; Figure 4I) consistent with our observation of increased variability in the frequency of spontaneous CaT events. There was no significant change in diastolic calcium levels (F0) between groups immediately following our 7-day ITPP protocol (*P* = 0.16; Figure 4J).

Optical mapping was also performed before and after administration of Arrythmia Inducibility Test (AIT; Figure 4K) in control and ITPP-treated hiPSC-aCM syncytia. Comparison of the difference in average baseline fluorescence during and after AIT serves to assess the change in diastolic calcium levels following burst tachypacing. In post-rest potentiation (PRP) of CaTs, ITPP-treated hiPSC-aCMs exhibited a reduced upstroke slope (*P* = 0.0004; Figure 4L) and decreased amplitude (*P* = 0.0008; Figure 4M) relative to controls. ITPP-treated hiPSC-aCM syncytia also exhibited a significantly diminished reconstitution of diastolic calcium post-AIT, (*P* = 0.035; Figure 4N) consistent with observed alterations to PRP CaT dynamics.

### 3.4 hiPSC-aCM and haCF co-culture recapitulates characteristics of AF-associated atrial fibrosis and conduction slowing/block

Co-culture of purified and matured hiPSC-aCM with haCFs was conducted at different seeding density ratios of 100/0, 90/10, and 70/30 to explore fibroblast-induced electrophysiological and calcium-handling changes. Co-cultures maintained the seeding ratios following 7 days of culture (100/0: 0.96 ± 0.03:0.01 ± 0.001; 90/10: 0.84 ± 0.03:0.15 ± 0.01; 70/30: 0.72 ± 0.03: 0.34 ± 0.03; Figures 5A,B). To verify pro-fibrotic effects of haCF culture, Collagen III expression was measured using immunocytochemistry. The 70/30 ratio (hiPSC-aCM/haCF) exhibited the highest expression of collagen III, which was fibroblast-dependent (*P* < 0.0001; Figures 5C,D). Furthermore, expression of the upstream cytokine Transforming Growth Factor beta 1 (TGFβ1) was also significantly increased in ratios consistent with increased collagen III expression (*P* = 0.0026; Figures 5E,F).

**Figure 5:**
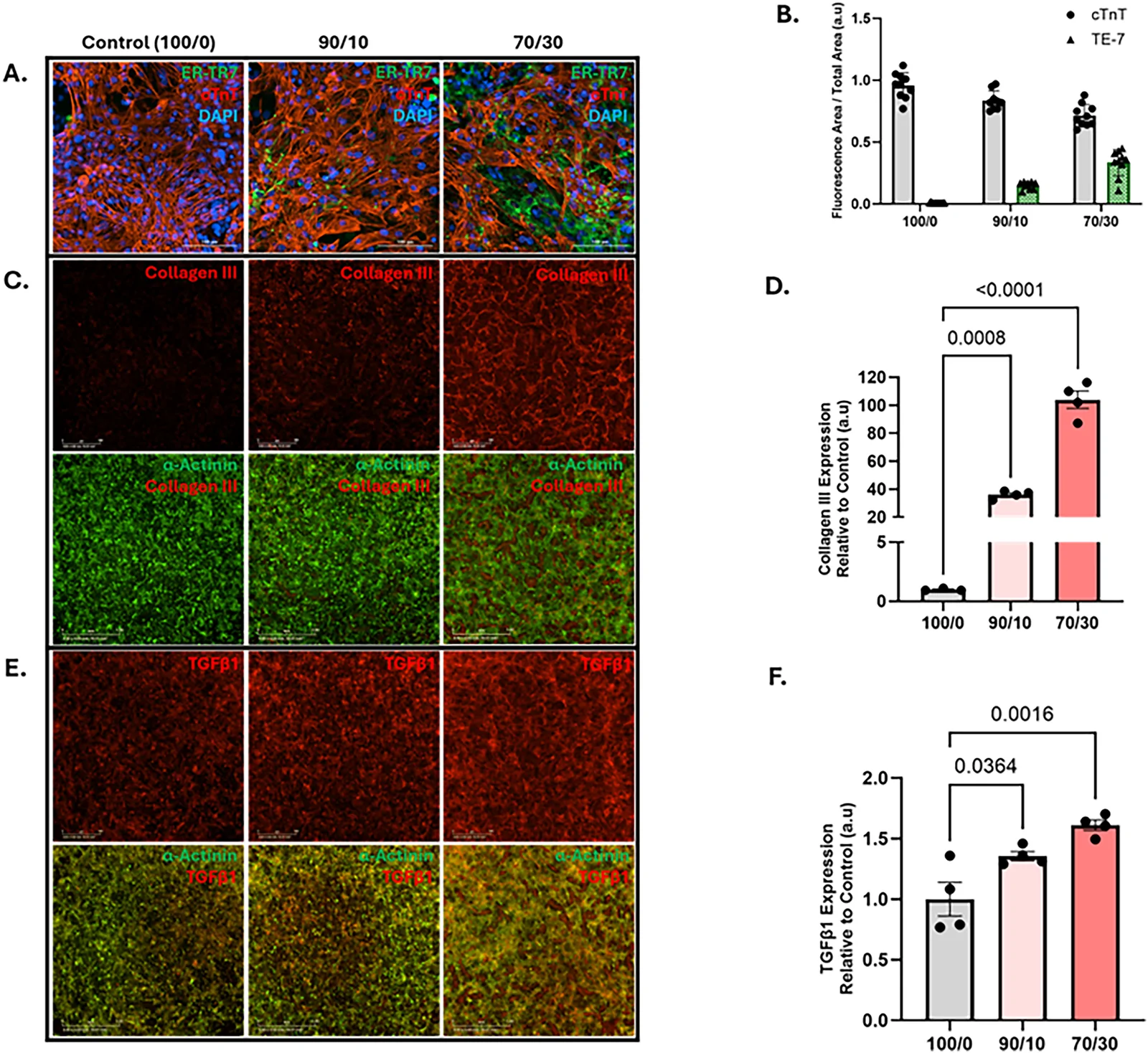
hiPSC-aCMs and haCFs co-culture induces atrial fibrosis. **(A)** Representative micrographs of cTnT/TE7 immunostaining in control (100/0) and co-culture (90/10, 70/30), and its **(B)** Fluorescence quantification after 7 days of co-culture with cTnT: TE7 for 100/0 (0.96 ± 0.03 to 0.01 ± 0.001), 90/10 (0.84 ± 0.03 to 0.15 ± 0.01) and 70/30 (0.72 ± 0.03 to 0.34 ± 0.03). **(C)** Representative micrograph panels of immunostaining for Collagen III **(C)** and TGFβ1 **(E)** with *α*-actinin labeling to highlight the cardiomyocyte fraction in the preparation. **(D)** Quantification shows a proportional increase with haCFs ratio in Collagen III **(D)** and TGFβ1 **(F)**.

Our previous reports demonstrated that fibroblast and non-cardiomyocyte co-culture affects electrophysiology of hiPSC-CMs in ratio-dependent fashion (27). We further expanded these studies into heart chamber-specific responses by subjecting hiPSC-aCM-haCF co-culture preparations to voltage and calcium optical mapping. Voltage mapping revealed a fibroblast-dependent decrease in spontaneous AP frequency, with representative traces shown in Figure 6A and quantitative analysis presented in Figure 6B (*P* < 0.0001). There was a concomitant prolongation of both the Fridericia-corrected AP duration at 80% repolarization (*P* < 0.0001; Figure 6C) and Fridericia-corrected APD triangulation (*P* < 0.0001; Figure 6D) at 70/30 ratio only. Furthermore, the upstroke slope of the AP (*P* < 0.0001; Figure 6E) and conduction velocity of the AP (*P* = 0.026; Figure 6F) were also decreased at 70/30 ratio.

**Figure 6:**
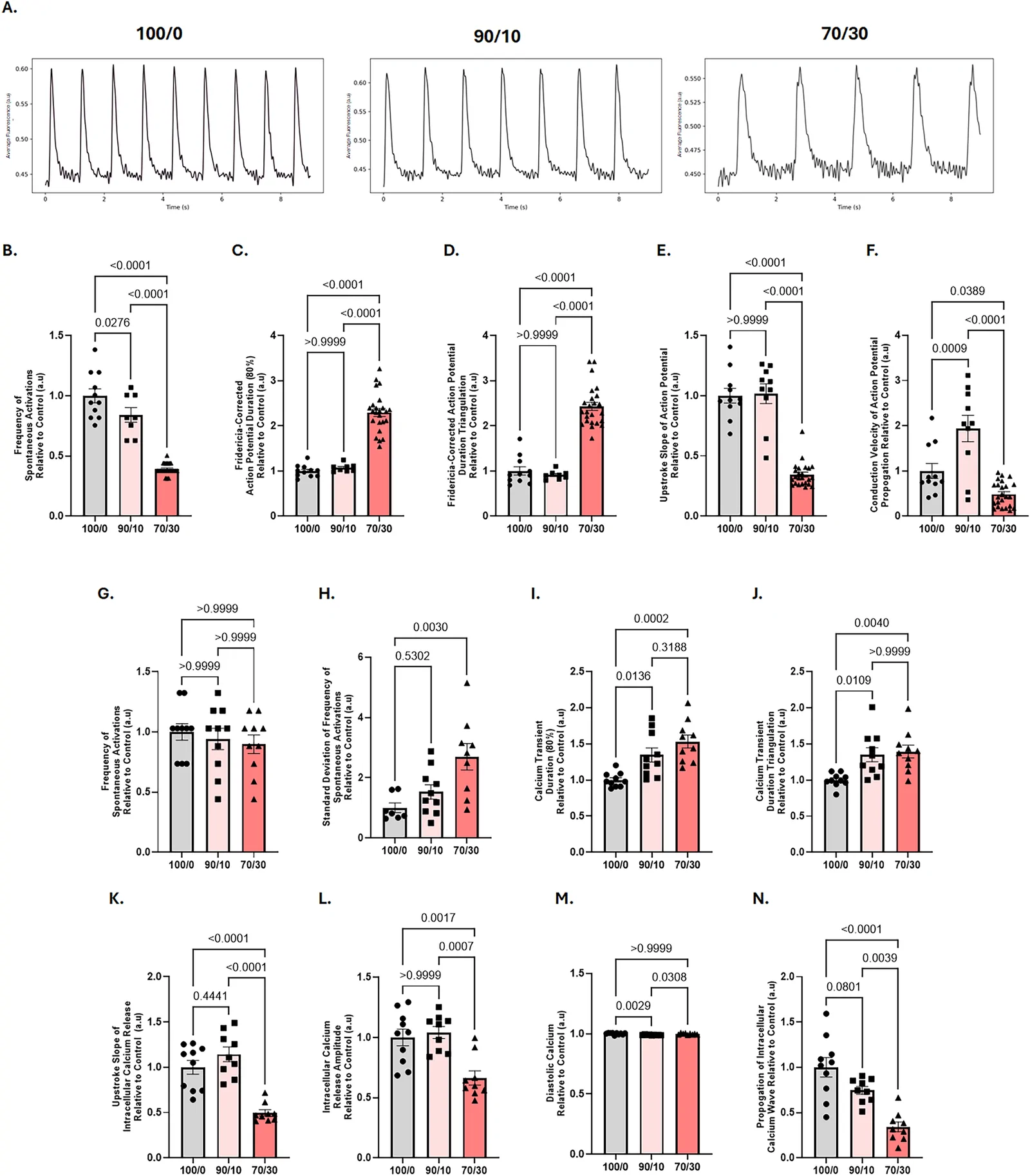
Fibrosis alters electrophysiology and intracellular calcium handling of hiPSC-aCM co-culture. **(A)** Representative time-sequence plots of action potential traces of control (100/0) and hiPSC-aCM/haCF co-culture at 90/10 and 70/30 ratios. Voltage mapping showed that: **(B)** spontaneous activation frequency decreased with increasing fibroblast proportion (90/10: Δ = −16 ± 9%, *P* = 0.0184; 70/30: Δ = −61 ± 6%, *p* < 0.0001). Co-culture with 30% haCFs further achieved prolongation of the Fridericia-corrected AP duration (80%) **(C)** (70/30: Δ = +130%±10%, *P* < 0.0001), prolongation of the Fridericia-corrected AP triangulation **(D)** (70/30: Δ = +143 ± 13%, *P* < 0.0001), and decrease in the upstroke slope of the AP **(E)** (70/30: Δ = −66 ± 6%, *P* < 0.0001) with conduction slowing **(F)** (70/30: Δ = −52 ± 18%, *P* = 0.0260). Calcium mapping indicated that: **(G)** No significant difference in the frequency of spontaneous calcium release events. **(H)** Prolongation of both the calcium transient duration (80%) (Δ = +53 ± 10%, *P* = 0.0002), and **(I)** duration of intracellular calcium reuptake was observed in ratio-dependent manner, most prominently at 70/30 co-culture (Δ = +39 ± 9%, *P* = 0.0022). **(J)** The diastolic calcium was decreased in 90/10 preparations (Δ = −1 ± 0.25%, *P* = 0.0028) without an effect at 70/30. **(K)** Significant increase in calcium release event variability at 70/30 co-culture (Δ = +170 ± 47%, *P* = 0.0030) with reduced in the upstroke slope **(L)** (Δ = −50 ± 8%, *P* < 0.0001), and amplitude **(M)** (Δ = −34 ± 9%, *P* = 0.0003) of intracellular calcium release. **(*N*)** Decrease in intracellular propagation of the calcium wave by 70/30 co-culture (Δ = −66 ± 12%, *P* < 0.0001).

Evaluation of co-culture intracellular calcium transients also demonstrated ratio-dependent changes observed to functional electrophysiology, particularly at 70/30 conditions. Co-culture did not affect the average frequency of intracellular calcium release events (*P* = 0.72; Figure 6G), however it prolonged both duration of CaTD_80%_ (*P* = 0.0001; Figure 6H), and the duration of calcium reuptake (*P* = 0.002; Figure 6I) in a ratio-dependent manner. Interestingly, 90/10 coculture presented a modest, significant decrease to diastolic calcium (*P* = 0.0019), however this was not recapitulated in 70/30 preparations (*P* = 0.77; Figure 6J).

Spontaneous intracellular calcium release frequency became markedly more variable at 70/30 ratio (*P* = 0.003; Figure 6K), with diminished intracellular calcium upstroke slope (*P* < 0.0001; Figure 6L) and intracellular calcium release amplitude (*P* = 0.001; Figure 6M). The propagation of the intracellular wave was significantly slower in 70/30 preparations (*P* < 0.0001; Figure 6N).

Given the similarities in the functional electrophysiology and calcium handling of 70/30 coculture to atrial fibrosis- a critical structural component of AF propagation, we hypothesized that coculture preparations subjected to electrical remodeling by ITPP would present conduction block analogous to this known proarrhythmic substrate. Voltage mapping of ITPP-treated co-cultures at 100/0 (Control; F-ITPP 100/0) and 70/30 (ITPP; F-ITPP 70/30) ratios verified that 70/30 preparations recapitulated tachycardia-mediated electrical remodeling observed in 100/0 iPSC-aCM syncytia (Figures 7A–C), including increased frequency of spontaneous activations (*P* = 0.028; Figure 7D) and shortening of the Fridericia-corrected APD_80%_ (*P* = 0.016; Figure 7E). Non-normalized spontaneous beating rates (not shown) composing Figure 7 consisted of Replicate 1: 100/0 Control: 1.16 ± 0.12 Hz, *n* = 4, 100/0 ITPP: 1.69 ± 0.14 Hz, *n* = 4, 70/30 ITPP: 1.33 ± 0.12 Hz, *n* = 4 and Replicate 2: 100/0 Control: 1.24 ± 0.03 Hz, *n* = 4, 100/0 ITPP:1.57 ± 0.06 Hz, *n* = 4, 70/30 ITPP: 1.68 ± 0.07 Hz, *n* = 4. The magnitude of change in frequency and APD80% was preserved between F-ITPP 70/30 or F-ITPP 100/0 conditions. Critically, F-ITPP 70/30 preparations did not demonstrate facilitated increase in the conduction velocity of the AP. Rather, conduction velocity was significantly decreased by inclusion of fibroblasts in F-ITPP 70/30 preparations in relation to F-ITPP 100/0 (*P* = 0.0001; Figure 7F).

**Figure 7:**
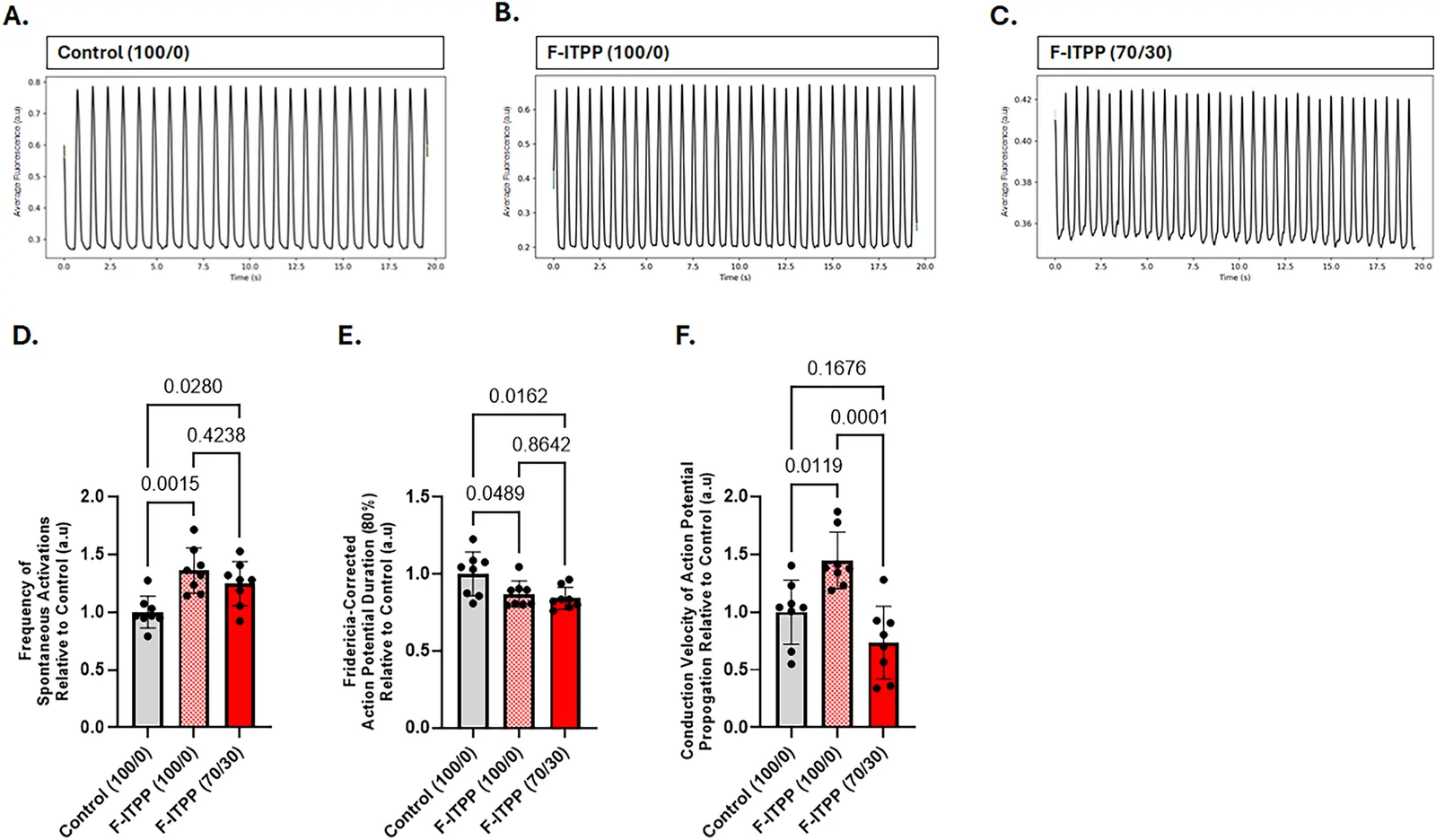
Fibrosis introduces a conduction block to intermittent tachypacing of hiPSC-aCMs. **(A–C)** Representative time-sequence plots of action potentials from control **(A)**, ITPP-treated 100/0 **(B)**, and **(C)** 70/30 hiPSC-aCM/haCF co-culture (F-ITPP). **(D)** Both 100/0 and 70/30 preparations responded to ITPP with increased frequency of spontaneous activations (F-ITPP 100/0: Δ = +36 ± 8%, *P* = 0.0015; F-ITPP 70/30: Δ = +25 ± 8%, *P* = 0.0280), and **(E)** shortened action potential duration (80%) (F-ITPP 100/0: Δ = −13 ± 6%, *P* = 0.0389; F-ITPP 70/30: Δ = −16 ± 6%, *P* = 0.0122). **(F)** F-ITPP 100/0 exhibited an increase to conduction velocity (F-ITPP 100/0: Δ = +45 ± 13%, *P* = 0.0089), while the F-ITPP 70/30 showed conduction slowing (F-ITPP 70/30: Δ = −27 ± 15%; *P* = 0.0001).

### 3.5 Molecular remodeling of calcium-handling and fibrotic signaling under intermittent tachypacing and fibroblast co-culture

To determine whether the electrophysiological and calcium-handling alterations observed under ITPP and fibrotic co-culture were associated with changes in calcium regulatory proteins, we analyzed the expression of phospholamban (PLN), phospho-phospholamban, pPLN(S16/T17), and sarco/endoplasmic reticulum Ca^2+^-ATPase (SERCA2a) by Western blot (Figure 8A). Total PLN expression was significantly reduced in both ITPP 100/0 (*P* = 0.0008) and ITPP 70/30 (*P* = 0.0006) (Figure 8B) compared with control conditions, whereas pPLN and SERCA2a protein levels remained unchanged (Figures 8C,D), consequently with preserved pPLN/PLN ratio (Figure 8E). Nevertheless, the (pPLN/PLN)/SERCA2a ratio was markedly elevated in the 70/30 ITPP group (*P* = 0.0022; Figure 8F), suggesting a cumulative effect of relative disinhibition of SERCA2a activity. This alteration in the PLN–SERCA2a regulatory balance is indicative of disturbed SR calcium reuptake dynamics and may underlie the variability in event-to-event spontaneous calcium release observed in the functional assays.

**Figure 8:**
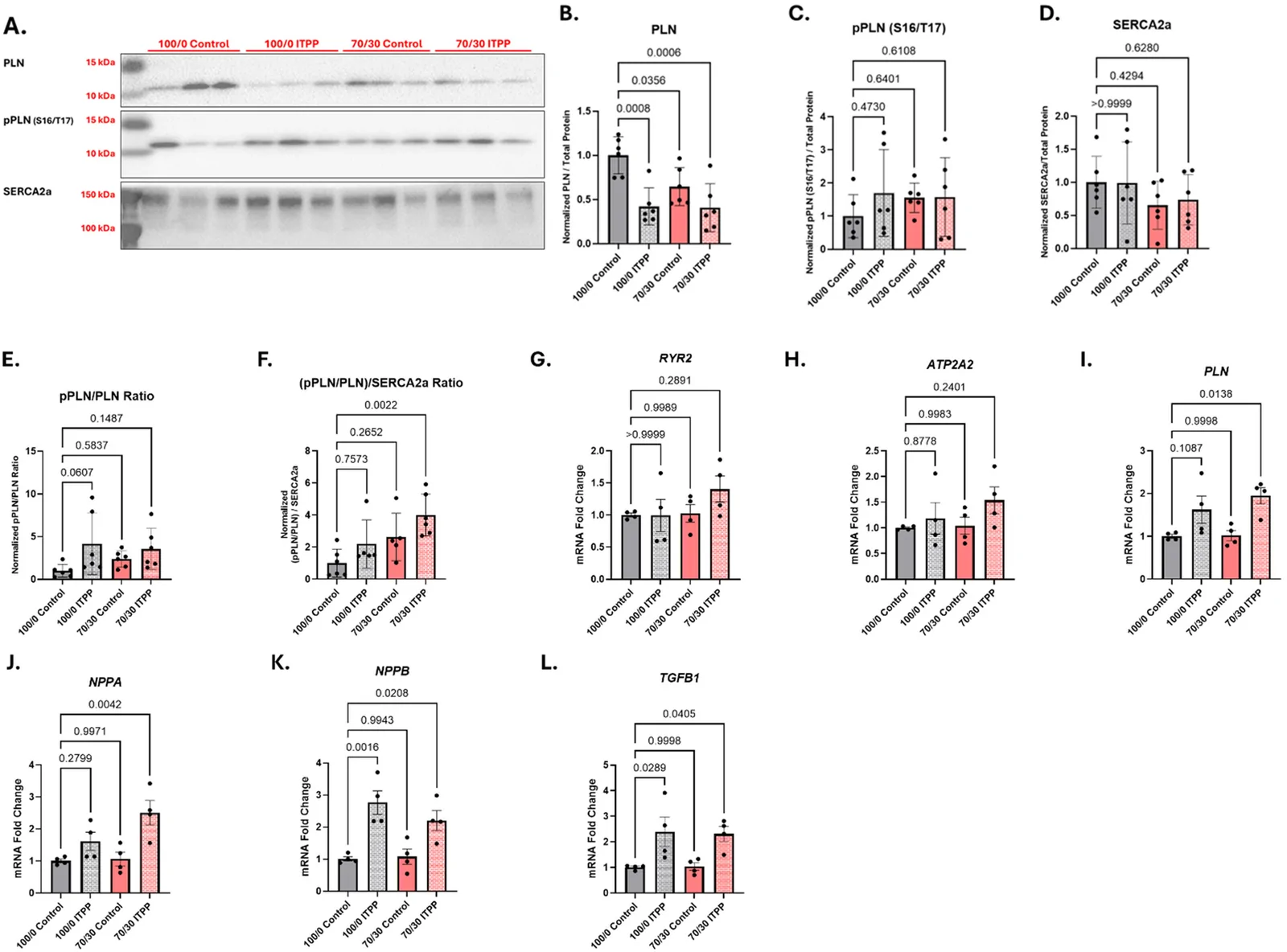
Molecular remodeling under intermittent tachypacing with fibrosis. **(A)** Representative western blot results for phospholamban (PLN) phospho-phospholamban (Serine-16 and Threonine-17; pPLN), and SERCA2a in control (100/0) and fibrosis (70/30) samples subjected to ITPP. Quantification of western blot results demonstrates significant downregulation of PLN across all 70/30 samples and 100/0 samples subjected to ITPP (100/0 ITPP: Δ = −57.88 ± 8.53%, *p* = 0.0008; 70/30 Control: Δ = −35.58 ± 8.79%, *p* = 0.0356; 70/30 ITPP: Δ = −59.29 ± 11.08%, *p* = 0.0006) **(B)**, without significant change to pPLN **(C)** and SERCA2a **(D)** expression, or the ratio of inactivated PLN (pPLN/PLN) **(E)** However, the summative ratio of inactivated PLN to SERCA2a was significantly increased when ITPP was applied to 70/30 fibrosis co-cultures (70/30 ITPP: Δ = +300.8 ± 53.46%, *p* = 0.0022) **(F)** Quantitative PCR (qPCR) analysis of calcium-handling and atrial fibrillation (AF)–related genes in hiPSC-aCM monocultures (100/0) and fibroblast co-cultures (70/30) under control and intermittent tachypacing (ITPP) conditions. Expression levels of *RYR2* (ryanodine receptor 2), *ATP2A2* (SERCA2a), *PLN* (phospholamban), *NPPA* (atrial natriuretic peptide A), *NPPB* (atrial natriuretic peptide B), and *TGFB1* (transforming growth factor β1) were normalized to housekeeping gene expression and expressed as fold change relative to control. In the 70/30 ITPP group, *PLN, NPPA*, *NPPB*, and *TGFB1* transcripts were significantly upregulated compared with their respective controls, whereas *RYR2* and *ATP2A2* remained unchanged. Uncropped blot images are provided in the Supplementary Figure S1.

At the transcriptional level, qPCR analysis showed that *RYR2* and *ATP2A2* expression remained unaltered across all conditions (Figures 8G,H), whereas *PLN* mRNA was significantly upregulated in the 70/30 ITPP group (*P* = 0.01; Figure 8I), suggesting a compensatory transcriptional response to altered SR calcium regulation. Atrial stress and remodeling markers *NPPA*, *NPPB*, and *TGFB1* were markedly increased following ITPP, with the most pronounced upregulation observed in the 70/30 co-culture condition, *p* = 0.004, *p* = 0.02, and *p* = 0.04 respectively (Figures 8J–L). The concurrent elevation of *TGFB1* and natriuretic peptides indicates activation of a pro-fibrotic and stress-responsive transcriptional regulation consistent with advanced atrial remodeling. Together, these molecular data demonstrate that intermittent tachypacing in an increased profibrotic signaling environment induces coordinated alterations in the PLN–SERCA2a axis and TGFβ1-driven signaling, recapitulating key aspects of calcium dysregulation and structural remodeling characteristic of atrial fibrillation.

## 4 Discussion

The aim of our study was to develop an improved modular human *in vitro* model that captures key features of AF-associated electrical remodelling and fibrosis using ECM matured hiPSC-aCMs and human adult atrial cardiac fibroblasts (haCFs). We assessed the chamber identity of the hiPSC employed in this work by comparing hiPSC-aCMs and hiPSC-vCMs calcium transients, oxygen consumption rate and gene expression. hiPSC-aCMs had a faster spontaneous beat rate than hiPSC-vCMs (Figures 1A,B) and shorter CaTD80% in comparison to -vCMs (Figure 1C). Further, we demonstrated that the faster spontaneous activity was associated with increased oxygen consumption rate in hiPSC-aCMs in comparison to -vCMs (Figure 1D). This characterization strongly corresponds to the functional phenotype of human atrial cardiomyocytes as published by our group and others (21, 24, 25). We further showed that the transcription of NPPA, MYL7 and KCNA5 were significantly higher in hiPSC-aCMs in comparison to -vCMs (Figures 1E–G). Although expression of KCNJ3 was higher in hiPSC-aCMs in comparison to vCMs, the difference was not statistically significant (Figure 1H). At last, NR2F2 expression was significantly lower in the hiPSC-aCMs in comparison to vCMs.

The results observed for NR2F2 might seem contradictory to the atrial identity of the cells; nevertheless, seminal work by Wu et al. (28) indicates that NR2F2 is necessary and sufficient to induce atrial specification during early embryogenesis but that the expression of this gene is highly time dependent and that later abrogation of NR2F2 expression did not alter nor revert established atrial identity. Further, Devalla et al. (29) confirmed that the expression of NR2F2 is highly dynamic and strongly induced by retinoic acid treatment, which was further supported by the work of Quaranta et al. (30). The latter also indicates that NR2F2 expression may be halted as cells progress into the path of maturation, which is the case for our cell cultures as evidenced in the new Figure 2 (confirmation of maturation). Also, RPMI/B27 maintenance medium does not contain retinoic acid, but retinyl acetate at a final concentration of 0.3 μM, which might not suffice to retain high levels of NR2F2 expression. The strong correspondence of hiPSC-aCM functional and transcriptomic data with the supporting literature, suggests that it is reasonable to conclude that the addition of retinoic acid to the differentiation protocol yielded atrial cardiomyocytes.

ECM-induced maturation of hiPSC-aCMs was further confirmed through increased TNNI3/TNNI1 expression as well as upregulated calcium handling proteins. Others have used electrical pacing of hiPSC-CMs without chamber specification to improve cardiomyocyte maturity in 2D and engineered heart tissues (31–33). Chronic electric pacing of 2D hiPSC-CMs cultures within a physiological range (60 beats/min) improved Ca^2+^ transient amplitude, upstroke velocity, and led to enlarged cell size with lower cell circularity (32). In hiPSC-CM engineered heart rings, chronic pacing at a constant supraphysiological rate (4 Hz) similarly improved contractility, structural and Ca^2+^ handling maturation (33). Although, hiPSC-aCMs operate at higher frequencies of activation in comparison to hiPSC-CMs, our attempts to continuously stimulate hiPSC-aCMs syncytia at 5 Hz for 7 days resulted in massive cell death and inability to conduct experiments (data not shown) and led to the decision to subject cells to intermittent tachypacing similarly to established models of atrial fibrillation (34–37).

In our study, the electrophysiological findings in ITPP-treated hiPSC-aCMs were consistent with previous studies showing tachycardia-induced electrical remodeling in both humans (38) and animal models of AF (39). Specifically, ITPP treatment led to a significant increase in spontaneous AP frequency and a shortening of APD in hiPSC-aCM syncytia. We did not observe significant changes to the AP upstroke slope under ITPP. Interestingly, optogenetic tachypacing approaches in both intermittent and continuous protocols noted an improvement to the AP upstroke slope (40, 41). This difference is likely methodological, as optogenetic pacing relies on inward permeability of channel rhodopsin Chrimson to monovalent cations (Na^+^, H^+^ and K^+^) that may directly influence depolarization kinetics. Furthermore, our ITPP model yielded a faster AP conduction velocity, which is a physiological mechanism to an increase in spontaneous frequency within the physiological range (from −1.5 Hz in control to −2 Hz under ITPP) reported in intact hearts (42).

In addition to the observed electrophysiological changes, significant alterations in calcium handling were identified in ITPP-treated hiPSC-aCMs. These included an increased variability in spontaneous calcium release events, reduced calcium release amplitude, and slower calcium wave propagation. Furthermore, upon application of AIT, ITPP-treated cells exhibited a reduced calcium upstroke slope and impaired reconstitution of diastolic calcium, which could contribute to conditions that predispose to calcium overload. It is known that DADs arise from spontaneous intracellular calcium release that can lead to premature action potentials hence contributing as a trigger for the onset of AF (43), which is replicated in our study.

Although we have previously demonstrated that cardiac fibroblasts can be differentiated from hiPSCs (44) (hiPSC-CFs), we were uncertain of the chamber identity of hiPSC-CFs and the potential impacts of this identity (or lack of) could have on cell-to-cell interactions. We reported previously that dermal fibroblasts blunt an arrhythmogenic effect in comparison to adult cardiac fibroblasts and hiPSC-derived cardiac fibroblasts (44). Furthermore, dermal fibroblasts greatly reduce action potential conduction velocity in comparison to adult cardiac fibroblasts and hiPSC-CFs (44). Therefore, we opted to investigate atrial fibrosis by co-culturing hiPSC-aCMs and human adult atrial fibroblasts (aCFs). We co-cultured hiPSC-aCMs with aCFs for seven days to recapitulate the cues delivered to cardiomyocytes during fibrosis: namely, deposition of ECM by aCFs, activation of pro-arrhythmic signaling pathways by aCF-derived cytokines and coupling between cardiomyocytes and aCFs (45).

Our co-culture reproduced key cellular and molecular features associated with endomysial fibrosis of AF (46). The 70/30 ratio of hiPSC-aCMs to haCFs exhibited the most pronounced fibroblast-induced changes, including a significant upregulation of collagen III and TGFβ1, both of which are known to be involved in fibrosis and the proarrhythmic substrate in AF. The electrophysiological remodeling we observed in these co-cultures were reduced AP frequency and prolonged APD, which may reflect altered ion channel expression or gating, delayed repolarization kinetics, change in resting potential due to myocyte-fibroblast coupling, where fibroblasts may contribute as a current sink (47). Further, the slowed conduction velocity is consistent with the fibrotic remodeling seen in AF, where increased fibrosis can impair electrical conduction and lead to re-entrant circuits (48). Additionally, the changes in calcium handling, including prolonged CaTD_80%_, reduced calcium upstroke slope and amplitude of calcium release, suggest that fibroblast-induced alterations may contribute to the arrhythmogenic substrate in AF.

The co-culture experiments also revealed that the 90/10 ratio shows a modest decrease in diastolic calcium levels, suggesting that a lower fibroblast content may have subtler effects on calcium handling. In contrast, the 70/30 ratio co-cultures exhibited more pronounced alterations in calcium homeostasis, as previously mentioned. These findings underscore the importance of fibroblast-to-cardiomyocyte ratio in modulating the electrophysiological and calcium-handling properties of the cells, potentially offering insights into the influence of fibroblast proliferation in addition to fibroblast identity in AF susceptibility.

To model advanced stages of AF, we combined the previous modules of fibrosis and ITPP into a single model by submitting cell cultures with endomysial fibrosis characteristics to intermittent tachypacing to obtain an entourage effect of proarrhythmogenic cues experienced by AF patients. We observed that ITPP of 70/30 (hiPSC-aCMs/haCFs) co-cultures exhibited a marked increase in the frequency of spontaneous activations and a shortening of APD. Also, a significant decrease in conduction velocity was observed, suggesting that fibroblast presence exacerbates the conduction block and electrical instability induced by tachypacing. The comparable AP frequency and APD observed in the F-ITPP 100/0 and F-ITPP 70/30 co-culture conditions suggest that fibroblasts do not further accelerate spontaneous activity and may primarily contribute to conduction slowing and structural heterogeneity.

Our Western blot analyses demonstrated a marked downregulation of PLN across both 100/0 and 70/30 conditions subjected to ITPP, without significant changes in pPLN(S16/T17) or SERCA2a protein levels. Notably, the ratio of inactivated PLN to SERCA2a was significantly elevated in the 70/30 ITPP group, which may indicate a relative shift toward SERCA2a disinhibition driven by decreased total PLN. Such maladaptive modulation of SERCA2a activity has been previously implicated in AF and heart failure, where reduced PLN phosphorylation or altered stoichiometry promotes intracellular calcium instability and triggers delayed afterdepolarizations (49, 50). Hoit et al. observed a similar pattern of PLN-SERCA2a homeostasis in canine atrial myocardium subjected to tachypacing, wherein total PLN protein expression decreased without change in SERCA2a levels (54). More recently, mechanisms of post-transcriptional PLN degradation in murine heart failure have also been elucidated, wherein PLN mRNA remained unchanged (55). While we observed an increase in PLN mRNA during ITPP, the relative cardiac mRNA may not reflect the bioavailable PLN protein pool- a hypothesis shared by other studies of failing human atrial myocardium (56). The ITPP model may therefore facilitate further investigation into mechanisms defining the post-transcriptional regulation of PLN in the setting of human atrial fibrillation.

Furthermore, atrial stress and remodeling markers *NPPA*, *NPPB*, and *TGFB1* were significantly increased under ITPP, particularly in fibrotic co-cultures, indicating activation of a pro-fibrotic and stress-responsive gene modulation. The upregulation of *TGFB1* reinforces the role of fibroblast–cardiomyocyte interactions in perpetuating the fibrotic signaling cascade, thereby exacerbating conduction slowing and electrical instability. Elevated TGFβ signaling has been widely recognized as a central mediator of structural remodeling and atrial fibrosis in AF, promoting extracellular matrix deposition and gap junctional uncoupling (38, 51). Collectively, these molecular findings provide mechanistic support for the phenotypic remodeling observed in the combined ITPP-fibrosis model and underscore the contribution of the PLN–SERCA2a regulatory imbalance and TGFβ-driven fibrotic signaling in sustaining AF-like electrical remodeling.

Overall, the combination of ITPP and fibroblast co-culture with hiPSC-aCMs establishes a useful *in vitro* model for investigating the electrical and calcium-handling alterations underlying AF susceptibility. Insights gained from this study may clarify AF mechanisms, identify potential therapeutic targets, and offer a novel NAM that enables investigation of additional AF triggers such as sympathetic and parasympathetic imbalance. Furthermore, this model could be integrated into established frameworks like the Comprehensive *in vitro* Proarrhythmia Assay (CiPA) and the Japan iPS Cardiac Safety Assessment (JiCSA) (52, 53), thereby enhancing the specificity of proarrhythmic risk assessment and serving as a valuable NAM for testing new AF treatments.

### 4.1 Limitations

While this study addresses maturation of hiPSC-CMs, controlled heterogeneity, chamber specificity, and effective electrical remodelling of hiPSC-aCMs, it is important to acknowledge that other dimensions of the myocardium such as the presence of other cell types like macrophages, endothelial cells, smooth muscle cells, as well as sympathetic and parasympathetic neurons were not recapitulated in this *in vitro* model. As such, it recapitulates key local environmental cues relevant to atrial remodeling, while not capturing full systemic regulation present in the intact heart. For all co-culture experiments, iPSC-aCMs and haCFs were cultured at confluence of 500,000 cells per 3.14 of cm^2^ of restricted free space, with immunofluorescence used to estimate the relative areas of co-culture conditions. Methods to specifically characterize the final proportions of co-culture, such as through flow cytometry, were not explicitly performed.

Additionally, this study was not designed to incorporate patient-specific genetic predispositions to AF. Furthermore, while optical mapping provides a composite of actuation and concatenation of different ion channels and electrogenic transporters, additional current-specific electrophysiological analysis at the single-cell level would provide greater mechanistic granularity. Similarly, without continuous optical mapping of activation patterns during treatment with ITPP that lasts 7 days in, which is technically challenging, results presented in this manuscript are interpreted as network-effects of stimulation upon the hiPSC-aCM syncytia and co-cultures respectively and cannot ascertain electrical remodeling at the level of the individual cardiomyocyte. Furthermore, during the 1.5 Hz phase the cultures may have continued to beat spontaneously, that 1:1 capture at either stimulation frequency was not confirmed, and that the applied frequencies should be interpreted as the stimulation regimen rather than confirmed cellular activation frequencies. Nevertheless, lack of optical mapping monitoring during ITPP does not impact on the validity of the model, the network-effects of the treatment, and is out of the scope of this manuscript.

In our study, we did not evaluate time-matched control for 1.5 Hz continuous stimulation of hiPSC-aCM syncytia, therefore the contribution of isolated stimulation at 1.5 Hz vs. intermittent 1.5 and 5 Hz stimulation cannot be distinguished explicitly.

The relative contribution of SR load to calcium decay could not be completely resolved without caffeine-induced calcium release studies. Future studies will aim to incorporate these approaches, as well as transcriptomic and proteomic profiling, to further elucidate AF mechanisms.

## 5 Conclusions

In conclusion, our NAM revealed that intermittent tachypacing in hiPSC-aCM and fibroblast co-cultures triggers coordinated electrophysiological, structural, and molecular remodeling reminiscent of atrial fibrillation. Functional alterations, including impaired calcium handling and conduction disturbances, are underpinned by downregulation of phospholamban, and activation of pro-fibrotic pathways such as TGFB1 and natriuretic peptides. These results highlight the interplay between calcium dysregulation and fibrotic signaling in driving AF-like phenotypes and establish a versatile human-relevant NAM for mechanistic investigation and evaluation of therapeutic interventions.

## Data Availability

Datasets are available on request: the raw data supporting the conclusions of this article will be made available without undue reservation.
