# Supplementary material for: hiPSC-derived new approach methodology (NAM) recapitulates hallmarks of atrial fibrillation
Source: Front Cardiovasc Med. 2026 Oct 20;13:1814075. doi: 10.3389/fcvm.2026.1814075 (PMC13727422; doi:10.3389/fcvm.2026.1814075)
Supplement: Supplementary file 1 [file Datasheet1.pdf]

SUPPLEMENTARY MATERIAL

hiPSC-Derived New Approach Methodology (NAM) Recapitulates Hallmarks of Atrial Fibrillation

Paul Kozlowski[1a], Kalai Mangai Muthukumarasamy[2a], Tiago William Buranello[3,4], Sung Jo[3], Amrish Deshmukh[2], Andre Monteiro da Rocha[2, 3b], Hakan Oral[2, 3b]

[1]Medical School, University of Michigan, Ann Arbor, MI

[2]Internal Medicine – Cardiovascular Medicine, University of Michigan, Ann Arbor, MI

[3]Frankel Cardiovascular Center Cell Regeneration Core Laboratory, University of Michigan, Ann Arbor, MI

[4]Departmento de Cirurgia – Faculdade de Medicina Veterinaria e Zootecnia, Universidade de Sao Paulo

[a]Equal contribution

[b]Co-corresponding Authors

Authors Address:

Cardiovascular Center, SPC 5853,

1500 East Medical Center Dr,

Ann Arbor, MI 48109-5853

Email: moandre@umich.edu or oralh@umich.edu

METHODS

Atrial Cardiac Myocyte Differentiation of hiPSCs

Human iPSCs were cultured on multi-well plates coated with Matrigel (Corning) and maintained in either StemMACS iPS-Brew XF (Miltenyi Biotec) or mTESR Plus (STEMCELL Technologies) media. hiPSCs were differentiated into an atrial-specific lineage using a modified GiWi protocol (1). After the cells reach 90-100% confluency, on differentiation (day 0), Basal Medium (RPMI 1640 supplemented with L-Glutamine and 25 mM HEPES (Gibco), supplemented with 0.5 mg/mL BSA (Sigma), 0.2 mg/mL L-Ascorbic Acid (Sigma), and 4 μM CHIR99021 (Selleck) was added. 48 hours following CHIR99021 treatment (day 2), medium was changed to Basal Medium containing 5 μM IWP4 (Selleck). On day 3, medium was switched to Basal Medium containing 5 μM IWP4 and 1 μM Retinoic Acid (Selleck). On day 4, the medium was changed with Basal Medium containing 1 μM Retinoic Acid. On day 6, the medium was changed to Basal Medium without any small molecules. On day 8, the medium was switched to RPMI 1640 with L-Glutamine (Gibco) supplemented with B-27 (Gibco). After day 8, the medium was refreshed every 48 hours with RPMI 1640 with L-Glutamine supplemented with B-27 until cell purification.

Purification and maturation of hiPSC-aCMs

Human iPSC-derived atrial differentiations were purified by negative-selection using biotin-conjugated magnetic bead sorting with an autoMACS Pro Separator (Miltenyi). The cells were dissociated with 0.25% Trypsin/EDTA (Fisher). Following trypsinization, EB20 Medium (80% DMEM/F12, 0.1 mmol/L nonessential amino acids, 1 mmol/L L-glutamine, 0.1 mmol/L β-mercaptoethanol, 20% FBS and 10 μmol/L blebbistatin) with 1 μM ROCK inhibitor (ROCKi; Y-

27632, Tocris) was added. The cells were dissociated and then strained through a 70 µm cell strainer. Total cell count was estimated using a hemocytometer. The cell suspension was centrifuged at 1000 RPM for 5 minutes and the supernatant was aspirated. The cell pellet was resuspended in cold autoMACS Running Buffer (Miltenyi) and centrifuged again at 1000 RPM for 5 minutes. The cells were purified by depleting iPSC-derived non-cardiomyocytes by magnetic separation using Miltenyi isolation kit according to the manufacturer's instruction. Maturation of hiPSC-aCMs on MatrixPlus was facilitated as previously described (2-4). A 96-well black microclear plate was coated with MatrixPlus, then rehydrated with HBSS with calcium and magnesium at 37 °C for 30 mins. The purified hiPSC-derived atrial cardiomyocytes were replated at a density of 95,000 cells/well. The hiPSC-aCMs were initially cultured in EB20 Medium with ROCKi for 48 hours, after which the medium was replaced with RPMI 1640 with L-Glutamine, supplemented with B-27 for 5 days, with media changes every 48 hours. All experiments were performed 7 days post-purification, at which point hiPSC-aCMs had formed mature syncytia.

Oxygen consumption rate

Oxygen consumption rate (OCR) was determined with Resipher 32-sensors (Lucidlab, USA) continuous oxygem consumption monitoring system. Briefly, hiPSC-aCMs or -vCMs were plated on MatrixPlus coated 96 well plates and allowed to mature for 7 days prior to a 24 hours OCR determination. Resipher sterile lid equipped with ultra-sensitive optical probes were placed onto standard multi-well cell culture plates. These micro-probes perform continuous vertical micro-movements to measure the dissolved oxygen concentration gradient that naturally forms in the media as cells consume oxygen. A built-in signal processing algorithm then converts these high-resolution gradient readings into real-time oxygen consumption rates (OCR), which are securely

streamed to a cloud-based application for remote tracking. After 24 hours of data collection averaged OCR was normalized to cell confluency and expressed as (fmol/mm2/s)/confluence%.

Culture of haCFs

Cryopreserved human donor atrial cardiac fibroblasts (haCFs; NHCF- Human Cardiac Atrial Fibroblasts; Lonza, #CC-2903) were obtained from Lonza Biosciences (Walkersville, MD) and thawed into FBM^{TM} Basal Medium (Lonza, #CC-3131) supplemented with FGM^{TM}-3 SingleQuot Supplements (Lonza, #CC-4525). The cells were cultured in T-25 cell culture flasks coated with 0.1% gelatin (Type A, Sigma) in 1x PBS. The haCFs were maintained for 2-4 days until they reached 70-80% confluency, at which point they were passaged for experiments (not more than 5 passages) according to the manufacturer's protocol.

Genetically Encoded Calcium Indicator Transfection of hiPSC-aCMs

To enable long-term optical mapping of calcium transients, hiPSC-aCMs submitted to ITPP were transfected with the genetically encoded calcium indicator, GCaMP6 fast variant, as previously described (3). Recombinant adenoviruses (AdGCaMP6f, Vector Biolabs, Malvern, PA, #1910) were prepared and had a titer of 1×10^{10} PFU/mL. Human iPSC-aCMs were transfected with a load of 5 multiplicity of infection (MOI) GCaMP6f in 100 μL of RPMI with L-Glutamine and without phenol red supplemented with B-27 (Gibco) for 48 hours. Cells were further incubated in RPMI with L-Glutamine and without phenol red for 3 days to allow for endogenous expression of GCaMP6f; all transfection protocols were performed within a 7-day window prior to induction of ITPP.

Arrhythmia Inducibility Test

Following 7 days of intermittent-tachypacing, hiPSC-aCM monolayers were subjected to a point stimulation test with simultaneous optical mapping. Optical mapping of calcium transients (CaTs) was conducted by placing the 8-well plate beneath a high-speed CCD camera (200 fps, 80 x 80 pixels; Red-Shirt Little Joe, Scimeasure, Decatur, GA). A heating block was used to maintain physiological temperature (37 °C) during recordings. An emission filter (515 nm; Chroma) appropriate to the loaded GCaMP6f, was used in combination with blue LED illumination to capture spontaneous or electrically induced CaTs. Point stimulation of the monolayers was facilitated using a dual-pronged electrode coupled to a MyoPacer™ power bank (IONOptix). The electrode was placed in direct contact with the monolayers under the guidance from the CCD camera and the monolayers were paced at 3.5 Hz (25 V) for 30s. Optical mapping of CaTs began 5s prior to termination of point stimulation, and resultant spontaneous CaTs were then recorded for 15s. Data analysis was conducted with a custom software (Scroll5).

Immunocytochemistry

Cells were fixed with 4% paraformaldehyde for 20 minutes and permeabilized in 0.1% Triton-X100 (Fisher) for 10 minutes at room temperature. After permeabilization, cells were washed once with 1x PBS and incubated for 30 minutes at room temperature in a blocking solution of 1% normal donkey serum in 1x PBS containing 0.1% Triton-X100. Primary antibodies (Table 1) were diluted in the blocking solution and incubated with the cells for 1 hour at room temperature. Cells were then washed with 1x PBS twice. Secondary antibodies (Table 1) were then applied in the blocking solution and incubated for 30 minutes at room temperature in the dark. Cells were washed with 1x PBS prior to nucleic acid staining with DAPI in 1x PBS (1:1000) for 5 minutes. Additional washes with 1x PBS were performed. Imaging was performed using either a Cytation

5 Imaging Reader (Biotek), Incucyte SX5 (Sartorius), or a Nikon A1R confocal microscope (Nikon).

Real-Time Quantitative PCR (qPCR):

Samples collected for determination of chamber specificity and maturation collected 7 days after purification and at the end of ITTP protocol were processed for RNA extraction using the Nucleospin RNA II kit (Macherey-Nagel, 740955.250) following the manufacturer's instructions. RNA concentration was quantified using a Nanodrop spectrophotometer. Complementary DNA (cDNA) synthesis was carried out using the High-Capacity cDNA Reverse Transcription Kit (ThermoFisher Scientific, 4368814, USA). qPCR was performed using TaqMan Fast Advanced Master Mix (ThermoFisher Scientific, 4444557) on a QuantStudio 3 Real-Time PCR System (Applied Biosystems). Relative mRNA expression levels were determined using the comparative Ct ($2^{-\Delta\Delta Ct}$) method, normalized to the geometric mean of the housekeeping genes *18S rRNA*, *HPRT1*, and *GAPDH*. The following TaqMan probes (ThermoFisher Scientific) were used for this study: *RYR2* (Hs00181461_m1); *ATP2A2* (Hs00544877_m1); *PLN* (Hs00160179_m1); *NPPA* (Hs00383230_g1); *NPPB* (Hs00173590_m1); *TGFB1* (Hs00998133_m1); *MYL7* (Hs01085598_g1); *NR2F2* (Hs00819630_m1); *TNNI3* (Hs00165957_m1); *TNNI1* (Hs00913333_m1); *18S rRNA* (Hs99999901_s1); *HPRT1* (Hs99999909_m1) and *GAPDH* (Hs99999905_m1). Additional, Taqman probes were used to assess chamber identity and maturation state.

Western Blot Analysis:

hiPSC-aCM and hiPSC/haCF co-cultures were lysed in cold RIPA buffer (Thermo) treated with Halt™ Protease and Phosphatase Inhibitor Cocktail (Thermo). Lysates were combined with Laemmeli sample loading buffer (Thermo) with 10% β-Mercapoethanol and heated at 95°C for 5

minutes. Samples were then resolved using SDS-PAGE on a 4–20% MiDi gradient gel at 120 V for 90 minutes and transferred to nitrocellulose membranes using a Power Blotter semi-dry system (Invitrogen). Membranes were stained with No-Stain™ Protein Labeling Reagent (Invitrogen) and total protein quantification was imaged using a ChemiDoc Imaging System (Bio-Rad). Membranes were then washed with 0.1% Tween-20 in PBS and blocked with a solution of 0.1% Tween-20 and 5% non-fat dry milk in PBS for 60 minutes. Overnight incubation with primary antibodies was carried out at 4°C with constant agitation. Membranes were triple-washed with a solution of 0.1% Tween-20 in PBS and incubated with secondary antibodies at room temperature for 1 hour with constant agitation. Detection of protein bands was performed using Western Lightning Plus ECL reagent (PerkinElmer, Waltham, MA, USA) and visualized using the ChemiDoc Imaging System (ImageLab 6.1; Bio-Rad). Protein levels were quantified using Bio-Rad image analysis software and normalized to total protein levels from the same membranes. Blot images were not subjected to any adjustments in contrast or brightness. The pseudocolor display was set to an inverted monochrome in selected blots for presentation purposes. Details of the antibodies used are listed in Table 1. Original gels, whole membranes, and individual blots are presented in Supplementary Figure S1. Images provided within supplement were obtained by direct screenshot and exported within ImageLab 6.1 software without further manipulation or use of outside software.

Statistical Analysis

All data are presented as mean ± standard deviation (SD) and normalized to the corresponding control values. Mean differences (Δ) between groups are reported along with the standard error of the difference. Outlier analysis was performed using ROUT with 1% sensitivity. Outliers between interrelated measurements (dF/F0, F0, Upstroke Slope, Conduction Velocity; or

Frequency, CaTD80 / APD80, CaTDTri / APDTri) were removed. For comparison of 2 groups, Kolmogorov-Smirnov test of normality was performed with subsequent unpaired parametric T-Test or Mann-Whitney U Test. For comparison of >2 groups, 1-Way-ANOVA was performed with Bonferroni comparison of individual groups to control, or Tukey’s test for multiple comparisons between groups. A two-tailed $P<0.05$ indicated statistical significance.

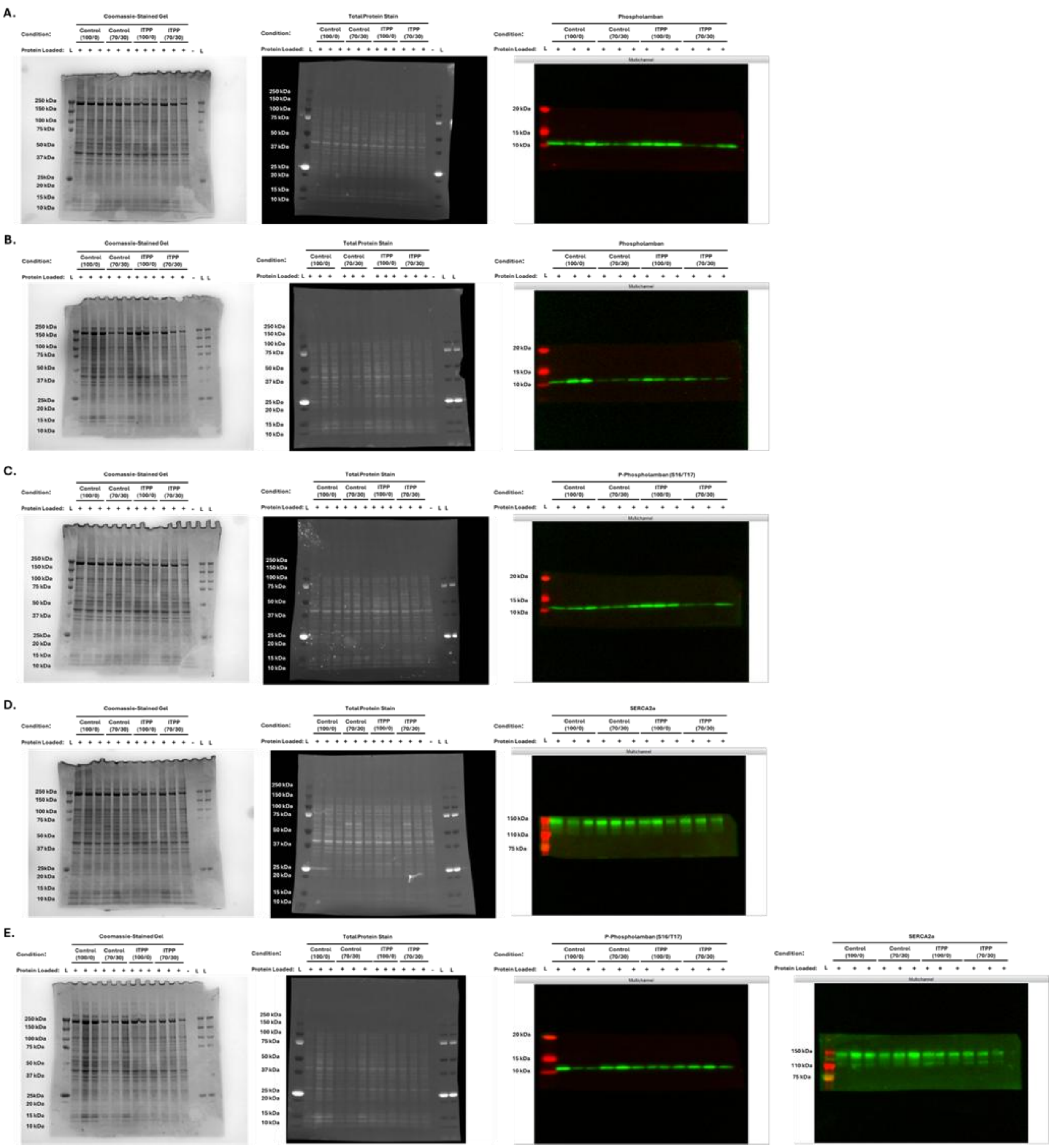

Supplementary Figure S1. Uncropped images of replicate blots for Phospholamban (A,B), p-Phospholamban (C,E), and SERCA2a (D,E) (for Figure 6) demonstrated alongside respective images of gels stained with Coomassie and whole-membranes stained for total protein. Image

labels depict experimental condition (Control or ITPP, 100/0 or 70/30) and corresponding identity of the protein load within each lane (“L” = ladder, “+” = sample, “-” = empty lane).

Table 1: List of antibodies utilized in this study

Antibody	Company	Catalog #	Species	Dilution
**Primary antibodies:**				
**Anti-TE-7 (ER-TR7)**	Millipore	CBL271	Mouse	1:100
**Anti-α-Actinin**	Sigma	A7811	Mouse	1:500
**Anti-cTnT**	Abcam	45932	Rabbit	1µg/mL
**Anti-TGFβ1**	Santa Cruz	sc-146	Rabbit	1:100
**Anti-Collagen III**	Invitrogen	PA5-34787	Rabbit	1:500
**Anti-SERCA2A**	Invitrogen	MA3-919	Mouse	1:500
**Anti-PLN**	Abcam	ab219626	Rabbit	1:2000
**Anti-pPLN (S16/T17)**	Invitrogen	702369	Rabbit	1:500
**Secondary antibodies:**				
**Donkey anti-Rabbit (594)**	Thermo Fisher	A21207	Anti-Rabbit	1:500
**Donkey anti-mouse (488)**	Jackson ImmunoResearch	715-545-150	Anti-Mouse	1:500

REFERENCES

1. Cyganek L, Tiburcy M, Sekeres K, Gerstenberg K, Bohnenberger H, Lenz C, et al. Deep phenotyping of human induced pluripotent stem cell-derived atrial and ventricular cardiomyocytes. JCI Insight. 2018;3(12).
2. Block T, Creech J, da Rocha AM, Marinkovic M, Ponce-Balbuena D, Jimenez-Vazquez EN, et al. Human perinatal stem cell derived extracellular matrix enables rapid maturation of hiPSC-CM structural and functional phenotypes. Sci Rep. 2020;10(1):19071.
3. Allan A, Creech J, Hausner C, Krajcarski P, Gunawan B, Poulin N, et al. High-throughput longitudinal electrophysiology screening of mature chamber-specific hiPSC-CMs using optical mapping. iScience. 2023;26(7):107142.
4. Monteiro da Rocha A, Allan A, Block T, Creech J, Herron TJ. High-Throughput Cardiotoxicity Screening Using Mature Human Induced Pluripotent Stem Cell-Derived Cardiomyocyte Monolayers. J Vis Exp. 2023(193).
